# Structural basis for the cyclophilin A binding affinity and immunosuppressive potency of *E*-­ISA247 (voclosporin)

**DOI:** 10.1107/S0907444910051905

**Published:** 2011-01-15

**Authors:** Andreas Kuglstatter, Francis Mueller, Eric Kusznir, Bernard Gsell, Martine Stihle, Ralf Thoma, Joerg Benz, Launa Aspeslet, Derrick Freitag, Michael Hennig

**Affiliations:** a F. Hoffmann–La Roche, Discovery Technologies, 4070 Basel, Switzerland; b Isotechnika Inc., 5120 75th Street, Edmonton, Alberta T6E 6W2, Canada

**Keywords:** voclosporin, cyclophilin A, *E*-ISA247, *Z*-ISA247

## Abstract

*E*-ISA247 (voclosporin) is a cyclosporin A analogue that is in late-stage clinical development for the treatment of autoimmune diseases and the prevention of organ graft rejection. The X-­ray crystal structures of *E*-ISA247 and its stereoisomer *Z*-­ISA247 bound to cyclophilin A have been determined and their binding affinities were measured to be 15 and 61 n*M*, respectively, by fluorescence spectroscopy. The higher affinity of *E*-ISA247 can be explained by superior van der Waals contacts between its unique side chain and cyclophilin A. Comparison with the known ternary structure including calcineurin suggests that the higher immunosuppressive efficacy of *E*-­ISA247 relative to cyclosporin A could be a consequence of structural changes in calcineurin induced by the modified *E*-­ISA247 side chain.

## Abstract 2

X-ray crystal structures of the cyclosporin A analogue *E*-ISA247 (voclosporin) and its stereoisomer *Z*-ISA247 bound to cyclophilin A suggest the molecular basis for the differences in their binding affinities and immunosuppressive efficacies.

## 1. Introduction

Cyclosporin A (CsA) is a cyclic undecapeptide fungal metabolite that is used for the prevention of acute rejection in solid-organ transplantation (Graeb *et al.*, 2004 ▶) and in the treatment of psoriasis (Sobell & Hallas, 2003 ▶). CsA exerts its immunosuppressive activity in a heterodimeric complex with the peptidyl–prolyl *cis*–*trans* isomerase cyclophilin A (CypA; Hamawy, 2003 ▶). Once formed, this complex binds to and inhibits the calcium-dependent phosphatase calcineurin (Cn). This prevents dephosphorylation of the transcriptional activator nuclear factor of activated T cells and its translocation from the cytoplasm to the nucleus. Consequently, the pro­duction of many immunosignalling cytokines is blocked and the immune reaction is suppressed (Hamawy, 2003 ▶). However, the long-term use of CsA is limited by side effects, notably nephrotoxicity (Burdmann *et al.*, 2003 ▶; Serkova *et al.*, 2004 ▶; Zachariae *et al.*, 1997 ▶).

The first X-ray structure of the CsA–CypA complex was reported at 2.8 Å resolution from tetragonal crystals (Pflugl *et al.*, 1993 ▶, 1994 ▶). Subsequently, CsA–CypA structures from ortho­rhombic (Mikol *et al.*, 1993 ▶) and monoclinic (Ke *et al.*, 1994 ▶) crystals that diffracted to 2.1 Å resolution were published. In addition, several crystal structures of CypA complexed with different CsA derivatives and linear peptide ligands have been reported (Taylor *et al.*, 1997 ▶). Finally, X-ray crystal structures of the ternary Cn–CypA–CsA complex were solved at 2.8 and 3.1 Å resolution (Huai *et al.*, 2002 ▶; Jin & Harrison, 2002 ▶). Analysis of the CsA–Cn interface suggested that modification of the butenyl group of the first CsA residue 4-­[(*E*)-2-butenyl]-4,*N*-dimethyl-l-threonine (Bmt1) might optimize its fit against the hydrophobic Cn surface and therefore increase the binding affinity.

**Figure 1:**
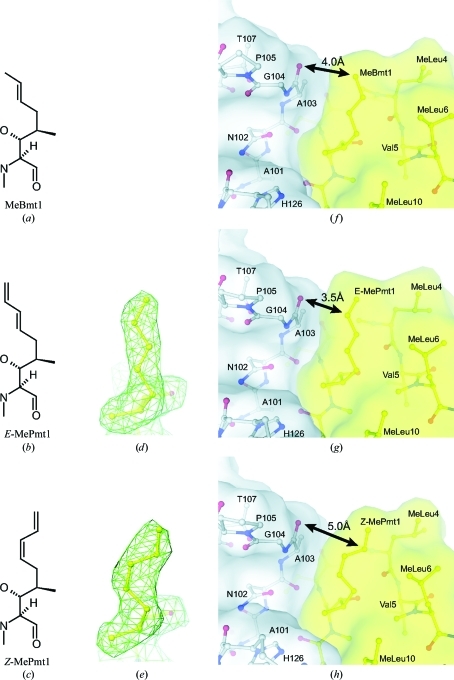
Residues at position 1 of CsA and the ISA247 stereoisomers. Structural formulae are displayed for (*a*) the cyclosporin residue MeBmt1, (*b*) the *E*-ISA247 residue *E*-MePmt1 and (*c*) the *Z*-ISA247 residue *Z*-MePmt 1. 2*F*_o_ − *F*_c_ electron-density maps around (*d*) *E*-MePmt1 and (*e*) *Z*-MePmt1 contoured at one standard deviation above the mean density are displayed in green. Surface and ball-and-stick representations of the crystal structures of CypA in grey complexed with (*f*) CsA (PDB entry 2rma; Ke *et al.*, 1994 ▶), (*g*) *E*-ISA247 and (*h*) *Z*-ISA247 in yellow are shown. The distances between the Ala103 backbone carbonyl of CypA and residue 1 of the cyclic peptides are marked by black arrows.

In the continuous search for immunosuppressive com­pounds with improved efficacy and safety profiles, the racemic CsA derivative ISA247 has been developed (Aspeslet *et al.*, 2001 ▶). In the *E* isomer of ISA247 (*E*-ISA247; voclosporin) the native MeBmt1 (Fig. 1 ▶ *a*) is replaced by 4-[(2*E*,4*EZ*)-2,4-pentadienyl]-4,*N*-dimethyl-l-threonine (*E*-MePmt1; Fig. 1 ▶ *b*), while in the *Z* isomer (*Z*-ISA247) this residue is replaced by 4-­[(2*Z*,4*EZ*)-2,4-pentadienyl]-4,*N*-dimethyl-l-threonine (*Z*-MePmt1; Fig. 1 ▶ *c*). *E*-ISA247 shows a greater immunosuppressive activity than CsA *in vitro* (Birsan *et al.*, 2005 ▶) and in *in vivo* animal models of autoimmunity (Maksymowych *et al.*, 2002 ▶) and transplantation (Aspeslet *et al.*, 2001 ▶; Gregory *et al.*, 2004 ▶). Two Phase III trials investigating the use of *E*-ISA247 for the treatment of moderate to severe psoriasis, a Phase IIb trial investigating the use of *E*-­ISA247 for the prevention of organ graft rejection following kidney transplantation and two Phase II/III trials investigating the use of *E*-ISA247 for the treatment of non-infectious uveitis have been completed (Bissonnette *et al.*, 2006 ▶; Dumont, 2004 ▶).

Here, we report the X-ray crystal structures of the CsA analogues *E*-­ISA247 (voclosporin) and *Z*-ISA247 bound to CypA. The equilibrium dissociation constants of both CypA–ISA247 complexes are determined and are rationalized by the observed structural data. The impact of the stereoisomer configuration of *E*-ISA247 on the binding interaction with Cn and the resulting immunosuppressive activity are discussed.

## 2. Materials and methods

### 2.1. Protein production

The human CypA gene was inserted into a pET21a plasmid and overexpressed in *Escherichia coli* BL21 (DE3) cells. A 20 l culture was grown at 303 K in LB medium and overexpression was induced at an optical density at 600 nm of 0.6 by adding IPTG to a final concentration of 0.5 m*M*. Cells were harvested after 6 h. 150 g biomass was resuspended in 750 ml of a buffer consisting of 50 m*M* MES pH 6.5, 10% saccharose, 5% glycerol, 10 m*M* MgCl_2_, 5 m*M* TCEP, 0.02% NaN_3_, 30 mg l^−1^ DNase I and four tablets of Complete protease-inhibitor cocktail (F. Hoffmann–La Roche) per litre. The cells were disrupted, the lysate was microfiltrated (0.2 µm) and concentrated by ultrafiltration (5 kDa) to about 200 ml and the buffer was exchanged by dia­filtration to 20 m*M* sodium phosphate pH 6.25, 2 m*M* TCEP, 5% glycerol, 0.02% NaN_3_ with four tablets of Com­plete protease-inhibitor cocktail (F. Hoffmann–La Roche) per litre. The protein solution was loaded onto a Macro-Prep Ceramic Hydroxyapatite Type I 20 µm column (Bio-Rad) and eluted with a gradient from 20 to 500 m*M* sodium phosphate. The pooled CypA fractions were dialysed against 20 m*M* MES pH 6.3, 1.5 m*M* TCEP, 0.02% NaN_3_, loaded onto a Source 15S column (Amersham Bio­sciences) and eluted with an NaCl gradient. Finally, CypA was purified on a Superdex 75 Prep-Grade (Amersham Bio­sciences) size-exclusion column equilibrated with 10 m*M* sodium/potassium phosphate pH 6.0, 100 m*M* NaCl, 2 m*M* TCEP, 0.02% NaN_3_.

### 2.2. Crystallization, X-ray data collection and model refinement

For CypA–ISA247 complex formation, CypA was concentrated to 50 mg ml^−1^ and mixed with 2 m*M* *E*-ISA247 or *Z*-­ISA247. The solution was incubated for 30 min at room temperature followed by 24 h on ice. The protein–peptide complexes were crystallized in hanging drops by vapour diffusion at 295 K using the same precipitant solution consisting of 20%(*w*/*v*) PEG 3350, 100 m*M* MES pH 6.5 and 200 m*M* ammonium sulfate. For X-ray data collection at 100 K, the crystals were transferred to precipitant solution containing an additional 20% glycerol and flash-frozen with liquid nitrogen. X-ray diffraction images of a CypA–*E*-ISA247 complex crystal were collected on a MAR345 image-plate detector (MAR Research) using Cu *K*α radiation from a Nonius FR591 rotating-anode X-ray generator. Images for CypA–*Z*-ISA247 crystals were collected on a 165 mm MAR CCD detector (MAR Research) using synchrotron radiation on the Swiss Light Source X06SA beamline. CypA–*E*-ISA247 complex data were processed with the *HKL* program suite (Otwinowski & Minor, 1997 ▶) to 2.2 Å resolution in space group *P*2_1_ and CypA–*Z*-ISA247 data were processed to 2.3 Å resolution in space group *P*2_1_2_1_2_1_ (see Table 1 ▶ for statistics). Molecular-replacement solutions were obtained with the program *MOLREP* (Vagin & Teplyakov, 2010 ▶) using the CypA–CsA crystal structure (PDB entry 2rma; Ke *et al.*, 1994 ▶) as a search model. Ten CypA–inhibitor complexes were identified in both space groups. Initial |*F*_o_| − |*F*_c_| maps calculated after rigid-body refinement against the CypA–*E*-ISA247 and CypA–*Z*-ISA247 data clearly showed the additional C atom of MePmt1 and the difference between its *E* and *Z* isomers. Both structures were refined by manual model fitting using the graphics program *MOLOC* (Gerber, 1992 ▶), model refinement using *REFMAC*5 (Murshudov *et al.*, 1997 ▶) and the placement of water molecules with *ARP*/*wARP* (Perrakis *et al.*, 1997 ▶). *B* factors were not restrained. 5% of the diffraction data were excluded from the refinement process for cross-validation. In the final models, the ten CypA–inhibitor complexes in the respective asymmetric units are essentially identical. The refinement statistics are summarized in Table 1 ▶. In the CypA–*E*-ISA247 structure Gly14 of chain *M* is poorly defined in the electron-density maps and is the only Ramachandran plot outlier. The coordinates and structure factors of the CypA–*E*-ISA247 and CypA–*Z*-ISA247 structures have been deposited in the Protein Data Bank with PDB codes 3odi and 3odl, respectively.

### 2.3. Fluorescence spectroscopy

**Figure 2:**
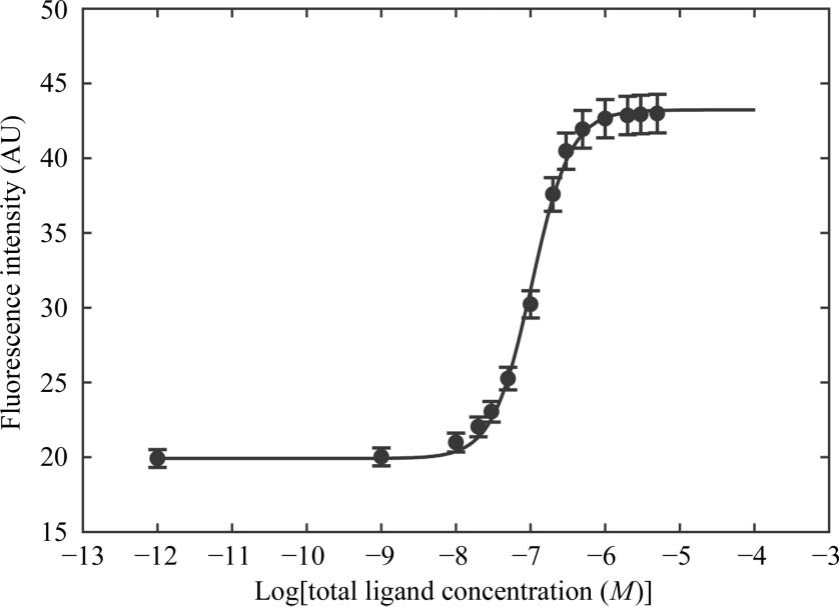
Fluorescence titration curve of the *E*-ISA247 isomer. The measured fluorescence intensities after correction for protein dilution (dots) are fitted with a sigmoidal curve (line).

Fluorescence measurements were performed on an SLM-Aminco 8100 double-grating spectrofluorometer using pre­viously described methods (Huber *et al.*, 2004 ▶). The intrinsic fluorescence of CypA was excited through its single tryptophan at 280 nm and was detected at 340 nm. The equilibrium dissociation constant *K*_d_ was determined by fluorescence titration at 200 n*M* protein concentration in 10 m*M* HEPES pH 7.4, 150 m*M* NaCl. Aliquots of *E*-ISA247, *Z*-ISA247 and CsA in DMSO were added to the protein solution (2 ml) and the resulting fluorescence intensities were measured. The final and constant DMSO concentration was 1.7%. The ISA247 isomers do not absorb at 280 nm and thus do not fluoresce. The measured fluorescence intensities were corrected for protein dilution (Birdsall *et al.*, 1983 ▶). A plot of the corrected fluorescence intensity *versus* ligand concentration was fitted with a sigmoidal curve (Fig. 2 ▶), from which the *K*_d_ was com­puted according to the law of mass action for a one-to-one protein–ligand complex (Eftink, 1997 ▶). The reported *K*_d_ values are averages from three titrations.

## 3. Results and discussion

### 3.1. ISA247 isomers do not induce major structural changes in the drug–CypA complex

The structures of CypA complexed with *E*-ISA247 and *Z*-­ISA247 were determined at 2.2 and 2.3 Å resolution, respectively, with excellent quality of the electron density. In both complexes, the structure of CypA, the backbone con­formation of the ISA247 isomers and the nonsubstituted ISA247 side chains 2–11 are essentially the same as in the structure of the CypA–CsA complex (Ke *et al.*, 1994 ▶). Significant differences between the three CypA–ligand structures are only observed in the side-chain conformation of residue 1 of the undecapeptides (Figs. 1 ▶ *f*–1*h*). The calculated electron-density maps clearly show the elongated shape of the MePmt1 *E* isomer (Fig. 1 ▶ *d*) and the bent shape of the *Z* isomer (Fig. 1 ▶ *e*). We conclude that the chemical modifications of the ISA247 isomers at residue 1 (Figs. 1*a*–1*c*) do not induce major re­arrangements of the CypA side chains in the drug–CypA complex and that functional differences between the two ISA247 isomers result from the structural difference at the modified MePmt1 residue only.

### 3.2. ISA247 stereoisomers differ in their interaction with CypA

We have determined the binding affinities of *E*-ISA247, *Z*-­ISA247 and CsA to its pharmacological binding partner CypA using fluorescence spectroscopy. The affinity of *E*-­ISA247, with a *K*_d_ value of 15 ± 4 n*M*, is essentially the same as the *K*_d_ of 13 ± 4 n*M* found for CsA. *Z*-ISA247, however, binds to CypA with an approximately fourfold higher dissociation constant (*K*_d_ = 61 ± 9 n*M*). This further strengthens the decision made early in the development of voclosporin to focus on the *E* isomer of ISA247 rather than to progress with the easier-to-produce racemate.

The only significant difference in the crystal structures of CypA complexed with *Z*-ISA247, *E*-ISA247 and CsA is observed in the side-chain conformation of the undecapeptide residue 1 (Figs. 1 ▶ *f*–1*h*). In the CypA–CsA complex MeBmt1 is positioned at the calcineurin-binding composite surface, between the CsA residues N-methylated leucine (MeLeu) 4, MeLeu6 and MeLeu10 on one side and the CypA residues Asn102, Ala103, Gly104 and His126 on the other side (Fig. 1 ▶ *f*). The MeBmt1 side chain is in van der Waals contact with the Ala103 backbone atoms. In the crystal structures of *E*-ISA247 (Fig. 1 ▶ *g*) and *Z*-ISA247 (Fig. 1 ▶ *h*) bound to CypA, the interaction patterns between the modified MePmt1 side chains and the Ala103 backbone differ from the pattern observed in the CypA–CsA structure (Fig. 1 ▶ *f*). While *E*-MePmt1 and MeBmt1 demonstrate high van der Waals complementarity with CypA, *Z*-MePmt leaves a gap between the protein and *Z*-ISA247. This is reflected in the distances of the CypA Ala103 carbonyl groups to the MeBmt1 (4.0 Å), *E*-MePmt1 (3.5 Å) and *Z*-MePmt (5.0 Å) CZ atoms in the respective structures. We suggest that lack of surface complementarity between CypA and *Z*-MePmt is the reason for the reduced affinity of the CsA analogue *Z*-­ISA247.

### 3.3. Impact of MeBmt1 modifications on Cn inhibition

**Figure 3:**
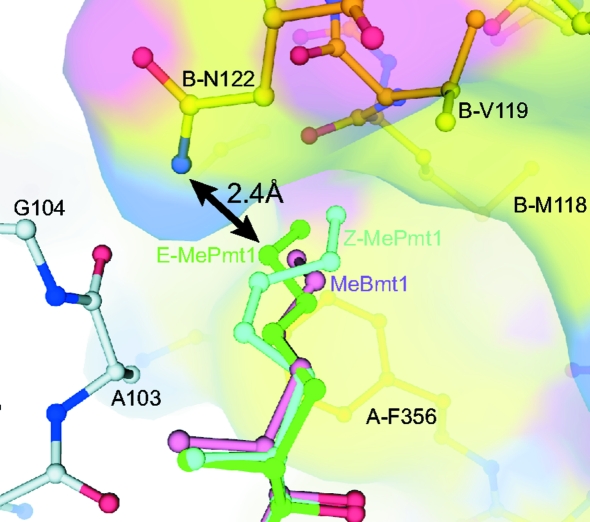
Superposition of the ternary Cn–CypA–CsA complex with the binary CypA–ISA247 complexes. Cn, CypA and the CsA residue MeBmt1 of the ternary complex (PDB entry 1m63; Huai *et al.*, 2002 ▶) are displayed in yellow, grey and pink, respectively. The MePmt1 residues of *E*-ISA247 and *Z*-ISA247 are shown in green and cyan, respectively. A close contact between *E*-ISA247 and Cn (carbon-to-nitrogen distance of 2.4 Å) is marked by a black arrow.

CsA binds to CypA and this heterodimeric complex binds to and inhibits calcineurin, inducing immunosuppression. The X-­ray crystal structures of the ternary Cn–CypA–CsA complex show that the binary CypA–CsA complex binds to a composite surface made up of residues from the Cn catalytic and regulatory subunits (Huai *et al.*, 2002 ▶; Jin & Harrison, 2002 ▶). In many cases the biological activity of CsA derivatives correlate with their affinity for CypA, but notable exceptions have been reported (Kallen *et al.*, 1998 ▶). In general, modifications at the CypA-binding residues 1, 2, 9, 10 or 11 that diminish binding to CypA also result in diminished immunosuppressive activity, while modifications of the effector-loop residues 4, 5 or 6 can strongly affect the immunosuppressive activity without significantly affecting CypA binding (Kallen *et al.*, 1998 ▶; Taylor *et al.*, 1997 ▶). *E*-ISA247 exemplifies an exception to this rule: it shows affinity for CypA comparable with that of CsA but superior immunosuppressive activity (Birsan *et al.*, 2005 ▶). To analyze the structural basis for this observation, we superimposed the CypA C^α^ atoms of the binary CypA–ISA247 complexes with the CypA C^α^ atoms of the ternary Cn–CypA–CsA complex and analysed the ISA247–Cn interfaces of the resulting Cn–CypA–ISA247 structure models (Fig. 3 ▶). Interestingly, the interaction patterns between Cn and the ISA247 isomers differ markedly from that observed with CsA. In the *E*-ISA247 ternary-complex superposition model the distance between the side chains of *E*-MePmt1 and the Cn residue Asn122 is only 2.4 Å. This demonstrates that at least one of these two amino acids has to adopt a significantly different conformation in order to avoid repulsion. Very limited conformational flexibility has been observed for the MeBmt1 side chain of CsA in the published CypA complex crystal structures, indicating that the Cn residue Asn122 is more likely to be affected. The modification of the first residue of *E*-ISA247 is unlikely to significantly change its conformational flexibility, in contrast to the modification of the fourth residue of the CsA analogue Debio 025, which not only limits the number of available side-chain conformations but also prevents binding of the Debio 025–CypA complex to Cn (Landrieu *et al.*, 2010 ▶). Asn122 is part of the so-called ‘latch region’ of the Cn regulatory subunit (Huai *et al.*, 2002 ▶; Jin & Harrison, 2002 ▶). This loop region is involved in activation of the Cn catalytic subunit and amino-acid sub­stitutions within this loop induce varying degrees of CsA resistance (Milan *et al.*, 1994 ▶). It has been suggested that modulation of Cn activity by CypA–CsA might be not only a consequence of spatial blockade of protein substrates but also of a direct involvement of CypA–CsA in the regulation of Cn activity (Huai *et al.*, 2002 ▶). This would provide one possible explanation for the differential immunosuppressive activities of the equipotent CypA binders *E*-ISA247 and CsA. Another possible explanation would be that the CypA–*E*-ISA247 complex binds to Cn with higher affinity as a result of the predicted structural changes at the Cn–*E*-ISA247 interface around residue *E*-­MePmt1.

## Supplementary Material

PDB reference: CypA–*E*-­ISA247, 3odi

PDB reference: CypA–*Z*-­ISA247, 3odl

## Figures and Tables

**Table 1:** Data-collection and refinement statistics Values in parentheses are for the highest resolution shell.

	*E*-ISA247	*Z*-ISA247
PDB code	3odi	3odl
Space group	*P*2_1_	*P*2_1_2_1_2_1_
Unit-cell parameters (Å, °)	*a* = 69.2, *b* = 161.3, *c* = 93.7, α = γ = 90.0, β = 100.1	*a* = 132.4, *b* = 141.1, *c* = 149.1, α = β = γ = 90.0
Copies per ASU†	10	10
X-ray source	Cu *K*α	SLS X06SA
Wavelength (Å)	1.5418	0.9799
Resolution range (Å)	25.0–2.2 (2.28–2.20)	40.0–2.3 (2.38–2.30)
No. of unique reflections	93907 (9188)	123151 (12158)
Completeness (%)	91.8 (88.0)	100.0 (100.0)
Multiplicity	3.3 (3.1)	9.5 (9.4)
Mean *I*/σ(*I*)	13.3 (2.6)	22.1 (6.7)
*R*_merge_‡	0.082 (0.399)	0.113 (0.424)
*B* factor from Wilson plot (Å^2^)	31.0	30.5
*R*_cryst_	0.164	0.169
*R*_free_ (%)	0.220	0.207
Root-mean-square deviations		
Bond lengths (Å)	0.008	0.007
Bond angles (°)	1.09	1.05
No. of protein residues/atoms	1644/12611	1650/12660
No. of ligand residues/atoms	110/860	110/860
No. of water molecules	1510	1726
Average *B* factor (Å^2^)		
All atoms	36.4	24.4
Water molecules	44.6	34.4
Ramachandran plot statistics, residues in (%)		
Favoured regions	96.2	96.4
Allowed regions	3.8	3.6
Outlier regions	0.1	0.0

† Number of CypA–ISA247 complexes per asymmetric unit in the crystal.

‡ *R*_merge_ = 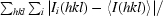 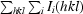.

## References

[bb1] Aspeslet, L., Freitag, D., Trepanier, D., Abel, M., Naicker, S., Kneteman, N., Foster, R. & Yatscoff, R. (2001). *Transplant. Proc.* **33**, 1048–1051. 10.1016/s0041-1345(00)02325-3 11267185

[bb2] Birdsall, B., King, R. W., Wheeler, M. R., Lewis, C. A. Jr, Goode, S. R., Dunlap, R. B. & Roberts, G. C. (1983). *Anal. Biochem.* **132**, 353–361. 10.1016/0003-2697(83)90020-9 6625170

[bb3] Birsan, T., Dambrin, C., Freitag, D. G., Yatscoff, R. W. & Morris, R. E. (2005). *Transpl. Int.* **17**, 767–771. 10.1007/s00147-004-0799-z 15827754

[bb4] Bissonnette, R., Papp, K., Poulin, Y., Lauzon, G., Aspeslet, L., Huizinga, R., Mayo, P., Foster, R. T., Yatscoff, R. W. & Maksymowych, W. P. (2006). *J. Am. Acad. Dermatol.* **54**, 472–478. 10.1016/j.jaad.2005.10.061 16488299

[bb5] Burdmann, E. A., Andoh, T. F., Yu, L. & Bennett, W. M. (2003). *Semin. Nephrol.* **23**, 465–476. 10.1016/s0270-9295(03)00090-1 13680536

[bb6] Dumont, F. J. (2004). *Curr. Opin. Investig. Drugs*, **5**, 542–550. 15202729

[bb7] Eftink, M. R. (1997). *Methods Enzymol.* **278**, 221–257. 10.1016/s0076-6879(97)78013-3 9170316

[bb8] Gerber, P. R. (1992). *Biopolymers*, **32**, 1003–1017.

[bb9] Graeb, C., Arbogast, H., Guba, M., Jauch, K. W. & Land, W. (2004). *Transplant. Proc.* **36**, 125–129. 10.1016/j.transproceed.2004.01.046 15041321

[bb10] Gregory, C. R., Kyles, A. E., Bernsteen, L., Wagner, G. S., Tarantal, A. F., Christe, K. L., Brignolo, L., Spinner, A., Griffey, S. M., Paniagua, R. T., Hubble, R. W., Borie, D. C. & Morris, R. E. (2004). *Transplantation*, **78**, 681–685. 10.1097/01.tp.0000131950.75697.71 15371668

[bb11] Hamawy, M. M. (2003). *Drug News Perspect.* **16**, 277–282. 10.1358/dnp.2003.16.5.829315 12942158

[bb12] Huai, Q., Kim, H.-Y., Liu, Y., Zhao, Y., Mondragon, A., Liu, J. O. & Ke, H. (2002). *Proc. Natl Acad. Sci. USA*, **99**, 12037–12042. 10.1073/pnas.192206699 PMC129394 12218175

[bb13] Huber, W., Perspicace, S., Kohler, J., Müller, F. & Schlatter, D. (2004). *Anal. Biochem.* **333**, 280–288. 10.1016/j.ab.2004.05.058 15450803

[bb14] Jin, L. & Harrison, S. C. (2002). *Proc. Natl Acad. Sci. USA*, **99**, 13522–13526. 10.1073/pnas.212504399 PMC129706 12357034

[bb15] Kallen, J., Mikol, V., Taylor, P. & Walkinshaw, M. D. (1998). *J. Mol. Biol.* **283**, 435–449. 10.1006/jmbi.1998.2108 9769216

[bb16] Ke, H., Mayrose, D., Belshaw, P. J., Alberg, D. G., Schreiber, S. L., Chang, Z. Y., Etzkorn, F. A., Ho, S. & Walsh, C. T. (1994). *Structure*, **2**, 33–44. 10.1016/s0969-2126(00)00006-x 8075981

[bb17] Landrieu, I., Hanoulle, X., Bonachera, F., Hamel, A., Sibille, N., Yin, Y., Wieruszeski, J. M., Horvath, D., Wei, Q., Vuagniaux, G. & Lippens, G. (2010). *Biochemistry*, **49**, 4679–4686. 10.1021/bi1003266 20423153

[bb18] Maksymowych, W. P., Jhangri, G. S., Aspeslet, L., Abel, M. D., Trepanier, D. J., Naicker, S., Freitag, D. G., Cooper, B. L., Foster, R. T. & Yatscoff, R. W. (2002). *J. Rheumatol.* **29**, 1646–1652. 12180723

[bb19] Mikol, V., Kallen, J., Pflugl, G. & Walkinshaw, M. D. (1993). *J. Mol. Biol.* **234**, 1119–1130. 10.1006/jmbi.1993.1664 8263916

[bb20] Milan, D., Griffith, J., Su, M., Price, E. R. & McKeon, F. (1994). *Cell*, **79**, 437–447. 10.1016/0092-8674(94)90253-4 7525078

[bb21] Murshudov, G. N., Vagin, A. A. & Dodson, E. J. (1997). *Acta Cryst.* D**53**, 240–255. 10.1107/S0907444996012255 15299926

[bb22] Otwinowski, Z. & Minor, W. (1997). *Methods Enzymol.* **276**, 307–326. 10.1016/S0076-6879(97)76066-X 27754618

[bb23] Perrakis, A., Sixma, T. K., Wilson, K. S. & Lamzin, V. S. (1997). *Acta Cryst.* D**53**, 448–455. 10.1107/S0907444997005696 15299911

[bb25] Pflugl, G., Kallen, J., Jansonius, J. N. & Walkinshaw, M. D. (1994). *J. Mol. Biol.* **244**, 385–409. 10.1006/jmbi.1994.1738 7990129

[bb24] Pflugl, G., Kallen, J., Schirmer, T., Jansonius, J. N., Zurini, M. G. & Walkinshaw, M. D. (1993). *Nature (London)*, **361**, 91–94. 10.1038/361091a0 8421501

[bb26] Serkova, N. J., Christians, U. & Benet, L. Z. (2004). *Mol. Interv.* **4**, 97–107. 10.1124/mi.4.2.7 15087483

[bb27] Sobell, J. M. & Hallas, S. J. (2003). *Semin. Cutan. Med. Surg.* **22**, 187–195. 10.1016/S1085-5629(03)00042-7 14649586

[bb28] Taylor, P., Husi, H., Kontopidis, G. & Walkinshaw, M. D. (1997). *Prog. Biophys. Mol. Biol.* **67**, 155–181. 10.1016/s0079-6107(97)00014-x 9446934

[bb29] Vagin, A. & Teplyakov, A. (2010). *Acta Cryst.* D**66**, 22–25. 10.1107/S0907444909042589 20057045

[bb30] Zachariae, H., Kragballe, K., Hansen, H. E., Marcussen, N. & Olsen, S. (1997). *Br. J. Dermatol.* **136**, 531–535. 9155953

